# A Qualitative Study Exploring the Experiences of Service Users With Complex Mental Health Needs

**DOI:** 10.1192/bjo.2022.94

**Published:** 2022-06-20

**Authors:** Pooja Saini, Laura Sambrook, Anna Balmer, Hana Roks, Jason McIntyre, Antony Martin, Jackie Tait, Peter Ashley-Mudie, Amrith Shetty, Rajan Nathan

**Affiliations:** 1 Liverpool John Moores University, Liverpool, United Kingdom; 2 QC Medica LLP, York, United Kingdom; 3 Cheshire and Wirral Partnership NHS Foundation Trust, Chester, United Kingdom

## Abstract

**Aims:**

Little is known about the experiences of individuals presenting with complex mental health needs and the provision of care they receive for suicide and self-harm behaviours. There are limited data describing the support individuals receive from services and, where they do, how this support is provided. Research suggests that those presenting with a more complex clinical presentation may have a history of both suicide attempts and self-harm. The aim of the study is to explore the experiences of individuals with complex mental health needs in respect of their self-harm and suicidal behaviours, and experiences of support received from mental health care services.

**Methods:**

A semi-structured interview methodology was used to generate qualitative data. Representative participants with complex mental health needs were recruited from across Cheshire and Wirral Partnership NHS Foundation Trust, UK. Ten participants were interviewed for the study. Interviews were audio-recorded and transcribed verbatim. A transcript-based conceptual analysis was conducted to identify and explore emerging themes.

**Results:**

The following three themes emerged from the service user interviews: (i) Service users discussed suicide attempts following inappropriate discharge; Service users spoke about feeling unsupported and not listened to by care staff, particularly as inpatients; and (ii) Service users expressed a necessity for staff training to improve understanding of self-harm and suicide attempts, having experienced negative consequences of staff handling when they may have self-harmed.

**Conclusion:**

This study highlighted the following recommendations for future suicide prevention for mental health services treating service users with complex mental health needs: increasing staff awareness of suicide or self-harm related issues; improving training and risk assessment skills; providing appropriate support for service users following discharge from inpatient settings; improving liaison and collaboration between services to provide better service user outcomes; and increasing awareness in listening to service users’ distress about suicidal or self-harm thoughts for each individual's situational context.

